# Sero-survey of bovine herpes virus-1 in dromedary camels and associated risk factors

**DOI:** 10.1186/s12917-022-03448-5

**Published:** 2022-09-30

**Authors:** Abdelfattah Selim, Salma Shoulah, Roua A. Alsubki, Fatima M. Albohairy, Kotb A. Attia, Itoh Kimiko

**Affiliations:** 1 Department of Animal Medicine (Infectious Diseases), Faculty of Veterinary Medicine, Benha University, Toukh, 13736 Egypt; 2 Department of Clinical Laboratory Science, College of Applied Medical Sciences, King Saud University, P.O. Box 2455, Riyadh, 11451 Saudi Arabia; 3 Department of Biochemistry, College of Science, King Saud University, P.O. Box 2455, Riyadh, 11451 Saudi Arabia; 4 Institute of Science and Technology, Niigata University, Ikarashi-2, Nishi-ku, Niigata, 950-2181 Japan

**Keywords:** *Bovine herpes virus-1*, Seroprevalence, Risk factor, Camels, Egypt

## Abstract

Infectious bovine rhinotracheitis (IBR) is a major animal health hazard in many countries throughout the world, caused by *bovine herpesvirus-1* (BoHV-1). The study’s goal was to evaluate the prevalence of BoHV-1 seropositivity among dromedary camels in three governorates in northern Egypt, as well as to identify risk variables related with BoHV-1 seropositivity. A total of 321 blood samples were collected randomly from dromedary camels living in the selected governorates and examined for presence of BoHV-1 antibody using ELISA test. The overall seroprevalence of BoHV-1 among examined camels was 5.92% (95%CI: 3.82–9.06). Univariable analysis confirmed that the significant association (*P* < 0.05) between sex, history of abortion, contact with small ruminants and herd size and BoHV-1 seropositivity. Using multiple logistic regression analysis, the following risk factors were identified to be related with the presence of BoHV-1 infection: sex (OR = 2.54, 95%CI: 0.63–10.22), history of abortion (OR = 4.16, 95%CI: 1.30–13.27), contact with small ruminants (OR = 5.61, 95%CI: 1.67–18.80) and large herd size (OR = 10.52, 95%CI: 2.46–44.91). This study estimated the disease’s seroprevalence in Egyptian dromedary camels, implying that camels could act as a BoHV-1 reservoir for transmission to other species.

## Introduction

Infectious bovine rhinotracheitis (IBR) is one of the most economically important diseases of ruminants with a global distribution [1]. *Bovine herpes virus-1* (BoHV-1) is the causal agent of IBR, is a member of the family *Herpesviridae* and belongs to the genus *Varicellovirus*, subfamily *Alphaherpesvirinae* [2]. BoHV-1 has three subtypes (BoHV-1.1, BoHV-1.2a, and BoHV-1.2b) that can cause a variety of disease forms, including respiratory infections (nasal discharges, respiratory manifestations and conjunctivitis), reproductive disorders in both males and females (balanoposthitis, vulvovaginitis and abortions), fever, loss of appetite, decreased milk yield and neonatal mortalities [3–5]. BoHV-1 infection can cause immunosuppression, which raises the risk of subsequent bacterial infections accompanying with respiratory infections [6, 7]. The virus transmitted mostly through inhalation or vaginal contact and can survive for a long period within populations because of its ability to become latent, reactivate, and quickly transmit among animals maintained in intense production units [8, 9].

Several studies in Egypt have reported the presence of BoHV-1 in cattle and buffaloes of different ages [10], but there are few records of the virus’s exposure in camels. Camels in Iran, Tunisia and Egypt have shown serological evidence of BoHV-1 exposure [11–13]. The virus was isolated from dromedary camels’ lung in Egypt and Sudan [14, 15], and was detected in llamas’ lungs in North America [16].

Egypt is a country with a low camel breeding population. However, large numbers of camels are imported each year from Sudan and Somalia for meat consumption [17]. Previous studies were provided that Sudanese camels are naturally exposed to BoHV-1, and the infection was confirmed using ELISA and FAT [15].

Therefore, this study investigates the epidemiology of BoHV-1 seropositivity in dromedary camels in some Egyptian governorates with large camel populations, as well as the risk factors related with BoHV-1 infection.

## Materials and methods

### Ethical statement

The Benha University ethical committee for animal studies approved all operations involving the handling and collection of blood samples (approval No: BUFVTM02–03-22). The owners of the camels gave their informed agreement and permission to collect the samples. The ethical committee at Benha University’s Faculty of Veterinary Medicine approved all procedures involving laboratory animals, which were carried out in compliance with current standards and regulations. The ARRIVE guidelines were followed when doing this research.

### Study area

**Fig. 1:**
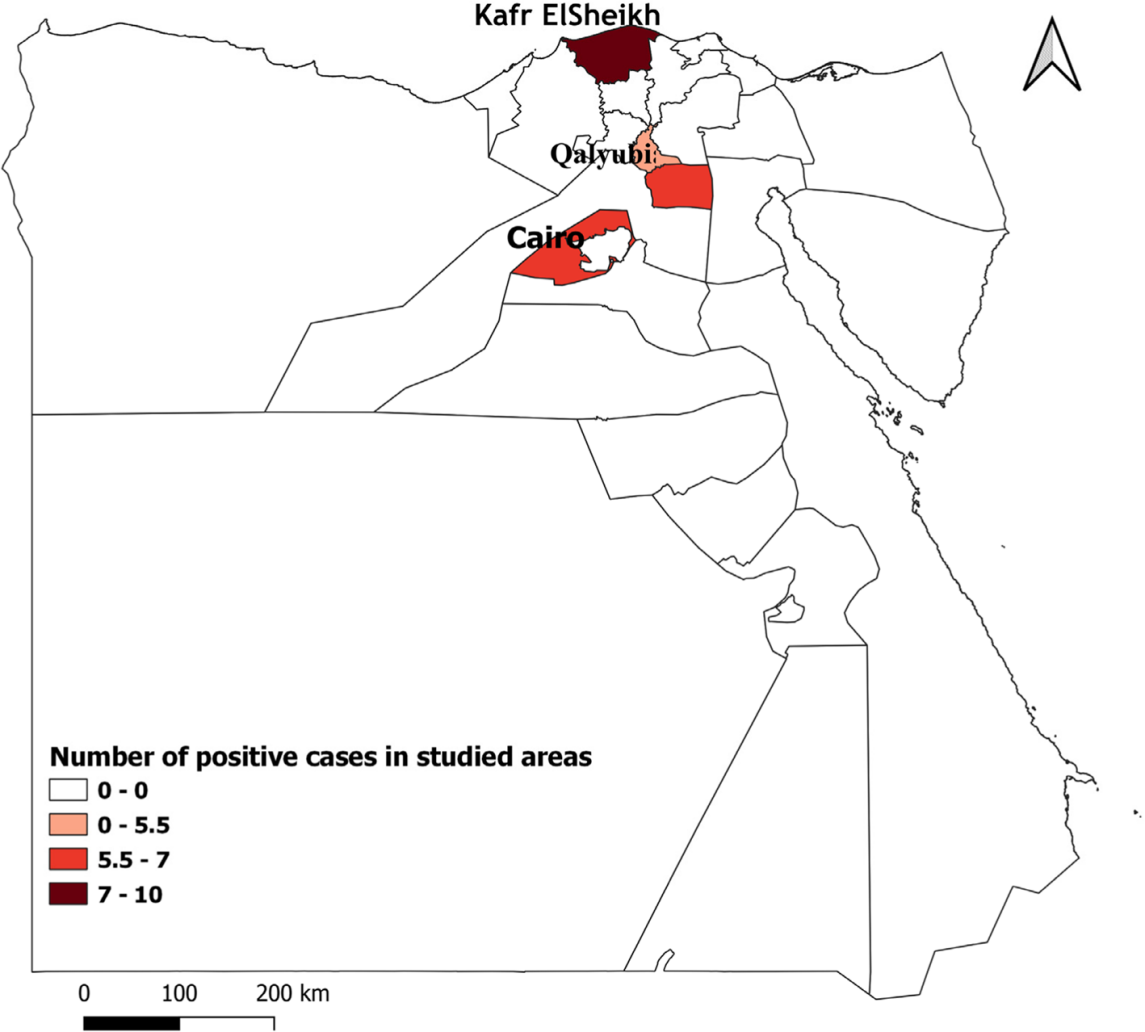
Map demonstrated the number of positive cases in each studied area

This study was conducted in three governorates in Northern Egypt (Cairo, Kafr ElSheikh, and Qalyubia). These governorates are geographically located at 30°02′N 31°13′E, 31.3°N 30.93°E and 30.41°N 31.21°E (Fig. 1). The climate is arid, desert climate, with summers that are lengthy, hot, humid, and dry, and winters that are cold, dry, and clearer. The temperature ranging from 25 to 40 °C in summer and 10–25 °C in winter.

### Sample size and samples collection

The minimum sample size was calculated using a random sampling approach using the Thrusfield formula [18], assuming a 2.8% of prevalence as previously reported by Eisa [12], a confidence level of 95, and 5% precision.

$$\mathrm{n}={\mathrm{Z}}^2\ \mathrm{P}\ \left(1-\mathrm{P}\right)/{\mathrm{d}}^2$$

Where n is the sample size, Z is standard normal distribution values (Z= 1.96), P is the predicted prevalence, and d^2^ is the absolute precision.

A total of 321 blood samples were collected randomly from camels living in three governorates at Northern Egypt. A total of 10 mL of blood was obtained through jugular venipuncture. Blood samples were stored at room temperature for 3 h to allow clotting before being centrifuged at 3.000 rpm for 15 minutes. The sera samples were separated and kept at − 20 °C until serological analysis.

### Animals

The sera were collected from free-ranging camels raising with sheep and goats, and these animals were used mainly for meat and milk production and breeding. Additionally, all camels included in this study were dromedary camels. An epidemiological questionnaire was used to analyse the potential risk factors for BoHV-1 in this study, which was administered to all the selected camel owners. The questionnaire was created to identify the most essential variables that could be related to BoHV-1 infection: 1) camels data such as sex (male, female), age (1–3, 4–9, 10–15 and > 15 years), and number of calving; 2) herd data like location, herd size (0–70, 71–140 and > 140), contact with small ruminant and history of abortion.

### Serological analysis

Serological investigation of antibodies against BoHV-1 in sera of sampled camels was performed using a competitive ELISA (ID Screen® IBR gB competition multi-species kits, IDVet, France) according to the manufacturer’s guidelines and protocols. The findings were represented as optical density (OD) and the absorbance was measured at 450 nm with an AllSheng ELISA microplate reader (AllSheng instrument Inc., Zhejiang, China). The proportion of inhibition of sample/negative control (S/N) was calculated to ascertain the result. If the S/N percent was equal to or less than 50%, the sample was declared positive for BHV-1gB.

### Statistical analysis

The data was analysed statistically using SPSS version 24 software (SPSS Inc., Chicago, IL, USA). Pearson’s Chi-square test was used to determine the association between BoHV-1 seropositivity and different risk factors (locality, sex, age, history of abortion, contact with small ruminants and herd size). All variables with *P*-value less than 0.25 in the univariate analysis were fitted for multivariate logistic regression models [19, 20]. Variables with *P*-value less than 0.05 estimated significant and included in the final model. The Hosmer and Lemeshow goodness-of-fit test was used to evaluate the model fit.

## Results

The percentage of camels with anti-BoHV-1 serum antibodies represented in Table 1. Out of 321 examined camels, 19 animals were seropositive, resulting in a prevalence of 5.92% (95%CI: 3.82–9.06). The prevalence rate of BoHV-1 in studied area showed non-disparity and the disease was mostly prevalent in Cairo (6.59%) and Kafr ElSheikh (6.84%) governorates. Univariate analysis was used to identify potential risk variables for BoHV-1 infection, Table 1. There was no correlation between seroprevalence of BoHV-1 in camels and age or calving number (*P* > 0.05). Moreover, the seroprevalence of BoHV1 increased significantly in females (9.68%) with history of abortion (13.48%), mainly in case of contact with small ruminants (9.80%), Table 1.

**Table 1:** Univariable analysis for variable associated with BoHV-1 seropositivity in dromedary camels

Variable	Total No of camels	No of positive (%)	95%CI	***P*** value
Locality				
Cairo	91	6 (6.59)	3.05–13.64	0.703
kafr ElSheikh	117	8 (6.84)	3.51–12.92	
Qalyubia	113	5 (4.42)	1.9–9.94	
Sex				
Male	166	4 (2.41)	0.94–6.03	0.006*
Female	155	15 (9.68)	5.95–15.36	
Age				
1–3	12	0	0–24.25	0.29
4–9	108	10 (9.26)	5.11–16.21	
10–15	147	7 (4.76)	2.32–9.5	
> 15	54	2 (3.70)	10.2–12.53	
History of abortion				
No	232	7 (3.02)	1.47–6.1	> 0.0001*
yes	89	12 (13.48)	7.88–22.1	
Number of calving				
non	211	10 (4.74)	2.59–8.5	0.348
1–2	62	6 (9.68)	4.51–19.55	
> 2	48	3 (6.25)	2.15–16.84	
Contact with small ruminants				
Yes	153	15 (9.80)	6.03–15.54	0.005*
No	168	4 (2.38)	0.93–5.96	
Herd size				
0–70	140	3 (2.14)	0.73–6.11	0.017*
71–140	103	7 (6.80)	3.33–13.37	
> 140	78	9 (11.54)	6.19–20.5	
Total	321	19 (5.92)	3.82–9.06	

*The result is significant at *P* < 0.05

In the final multivariable analysis, the factors associated with seropositivity for BoHV-1 infection shown in Table 2. Females were 2.54 times more likely than males to have BoHV-1 antibody. Also, the results proved that history of abortion (OR = 4.16, 95%CI: 1.30–13.27), contact with small ruminants (OR = 5.61, 95%CI: 1.67–18.80) and large herd size > 140 (OR = 10.52, 95%CI: 2.46–44.91) were associated with BoHV-1 seropositivity, Table 2.

**Table 2:** Multivariable logistic regression models for potential risk factors associated with BoHV-1 seropositivity in dromedary camels

Variable		B	S.E.	OR	95% C.I. for OR		***P*** value
					Lower	Upper	
Sex	Female	0.934	0.710	2.54	0.63	10.22	0.188
History of abortion	Yes	1.425	0.592	4.16	1.30	13.27	0.016
Contact with small ruminants	yes	1.724	0.617	5.61	1.67	18.80	0.005
Herd size	71–140	1.111	0.726	3.04	0.73	12.59	0.126
	> 140	2.353	0.741	10.52	2.46	44.91	0.001

*B* Logistic regression coefficient, *SE* Standard error, *OR* Odds ratio, *CI* Confidence interval

## Discussion

BoHV-1 is a globally distributed disease with significant geographical variation in incidence. Several researches have been conducted using serological surveys to detect risk variables for BoHV-1 seropositivity in ruminants [21–25] but few studies have been performed in camels especially in Egypt [12]. The present work identified the seroprevalence of BoHV-1 in camels in some Egyptian governorates and assessed the associated risk factors.

The detected seroprevalence for BoHV-1 antibodies in camels in this study was found 5.92%, which was lower than studies in other parts of Sudan of 76.9% [15] and Saudi Arabia of 13% [26]. The seroprevalence of this study lie in range of previous study from Tunisia of 5.8% [11] and Algeria of 3.7% [27] but higher than previous rate reported in Iran of 0% [13].

Interestingly, there is significant disparities in Egypt regarding BoHV-1 seroprevalence rates in camels. Two studies found greater rates of seroprevalence than our reported rate 14.2% [28] and 18.2% [29], while a third one found a lower rate of seropositivity 2.8% in farm samples and 2.1% in samples from abattoir [12].

The disparities in antibody seroprevalence observed in different locations and countries could be attributed to some factors such as sample size, analyze test, herd size differences, breeding practices, production systems, disease-control strategies, and age of camels [30–35].

The present findings revealed that the seroprevalence rate of BoHV-1 is significant higher in females especially with one to two calving. Similar results have been reported previously by Benaissa et al. [27] and Tadeg et al. [36]. This might be explained due to stress factors related to female as pregnancy, lactation or due to frequent exposure to infection from male during breeding [23, 37–39].

The findings are consistent with prior research, which found that the seroprevalence of BoHV-1 was strongly associated with abortion [36], they found that cow with history of abortion was at a risk to get BoHV-1 infection than non-aborted animals. Therefore, those results confirmed that the reproductive problem increase the risk of BoHV-1 infection in camels [40]. Although a positive ELISA test reveals that an animal was exposed to the BoHV-1 virus during its life, it does not prove that the virus was the cause of the abortion because it is not a direct diagnostic approach [41]. For such reasons, direct diagnostic procedures such as antigen detection ELISA or PCR would be necessary [42].

Moreover, in the line with previous findings of Benaissa, Youngs, Mimoune, Faye, Mimouni and Kaidi [27], the seroprevalence of BoHV-1 was more frequent among camels of median age group (4–9 years). This could be explained by repeated virus exposure over time, particularly in old animals or due to presence of persistent infected animals among studied animals [43–48]. In contrast, age of the animal has been identified as a risk factor for seropositivity of BoHV-1 in cattle [49, 50].

The studied camels had the chance to come into contact with small ruminants that were BoHV-1-positive due to the shared grazing area, which enhanced the risk of infection [51, 52]. Similar conclusion was reached by Benaissa et al. [27].

Importantly, a larger herd was substantially associated with a high prevalence of BoHV1 infection, which is consistent with earlier research of Solis-Calderon et al. [53]. They found that a larger herd, particularly one with a high population density, is linked to a higher prevalence of IBR.

## Conclusion

In Egypt, the BoHV-1 virus is circulating among camels, infecting a large number of animals. Contact with other small ruminants, sex, a history of abortion, and herd size are all risk factors for IBR infection in camels. Age and number of calving had not significant role in prevalence of the disease. More molecular and epidemiological investigations of BoHV-1 in camels are needed to assess the virus’s epidemiological and clinical implications in Egypt, estimate the true prevalence, and characterize the virus’s circulation.

## Data Availability

This article contains all of the data that was created or analyzed throughout the investigation.
